# The future of dementia risk reduction research: barriers and solutions

**DOI:** 10.1093/pubmed/fdw103

**Published:** 2016-10-03

**Authors:** S Mitchell, S H Ridley, R M Sancho, M Norton

**Affiliations:** Alzheimer's Research UK, Granta Park, Cambridge CB21 6AD, UK

**Keywords:** mental health, population-based and preventative services, research

## Abstract

**BACKGROUND:**

We examine why dementia prevention and risk reduction are relatively underfunded and suggest potential remediation strategies. The paper is aimed at researchers, funders and policy-makers, both within dementia and also the wider health prevention field.

**METHODS:**

A discussion-led workshop, attended by 58 academics, clinicians, funders and policy-makers.

**RESULTS:**

The key barriers identified were the gaps in understanding the basic science of dementia; the complex interplay between individual risk factors; variations in study methodology; disincentives to collaboration; a lack of research capacity and leadership and the broader stigma of the condition. Recommendations were made to encourage strategic leadership, provide greater support for grant applications, promote collaboration and support randomized control trials for the research field.

**CONCLUSION:**

Having identified the barriers, the key challenge is how to implement the potential solutions. This will require engagement with decision-makers within funding, policy and research to ensure that action takes place.

## Context

The world faces a growing global epidemic of dementia and as yet there are no therapies that delay or slow the progression of the condition. Up to 30% of Alzheimer's disease (AD)^1^ and more broadly, dementia^2^ cases may be preventable through modifiable health and lifestyle factors. Based on current evidence,^3^ key factors include low education in early life, hypertension in midlife, and smoking and diabetes throughout life. Interventions to address these factors will vary depending on the factor, but could include public health messaging, specialist support for smoking cessation or pharmacological management for diabetes.

Data from recent studies^4–6^ suggest that the age-specific prevalence of dementia in some European countries has either fallen or stabilized, potentially in response to changing health and lifestyle habits. However, these welcome benefits may be offset by burgeoning obesity and type II diabetes, which recent evidence suggests could increase dementia risk.^1^

The profile of dementia risk reduction has risen up the international policy agenda, highlighted by recent governmental and international attention through the World Dementia Council,^7^ and the Blackfriars Consensus Statement^8^ within the UK. However, throughout all these initiatives, there is a clear recognition that the evidence base to support policy decisions is incomplete and there remain many gaps in understanding, particularly in relation to how potentially modifiable risk factors contribute to the pathophysiology of diseases that result in dementia.^3^

The political focus on risk reduction is welcome, but serves to reveal the relative lack of funding in this area. Estimates suggest that ~5% of the dementia research portfolio in the UK is associated with prevention and risk reduction research,^9^ although it should be noted that this is consistent across the spectrum of biomedical research into other diseases.^10^ The International Alzheimer's Disease Research Portfolio database reports that 26 out of 1197 grants (2%) in 2014 were on primary prevention or risk reduction.^11^

## Methods

This paper reflects the collective thinking of 58 leaders in dementia research invited to a 1-day workshop by Alzheimer's Research UK, who represent key contributors in their field. There was input from researchers, clinicians, funders and policy-makers from both the UK and internationally; the acknowledgements section lists all attendees. The workshop commenced with a series of key note presentations, followed by facilitated discussions with scribes at each table to capture points raised. We sought to identify the reasons for the historic and current paucity of research funding for dementia risk reduction by considering the following key questions:

1. Are the right research questions being asked when developing research studies?
2. Is there sufficient underpinning knowledge to be able to frame appropriate research questions?
3. Are there particular challenges around methodology, technology or infrastructure?
4. Do appropriate funding opportunities exist?

At the end of the workshop, consensus was sought on the key barriers and most important solutions. These findings formed the basis of this paper. This approach enabled us to develop conclusions that reflect views from across the field, and to build on existing views of the current research climate.^3,8^

## Findings

These findings are aimed at researchers, funders and policy-makers. We hope that researchers will apply this analysis to their study designs and that funders will build many of these suggestions into their decision-making processes. We recognize that there is wider public interest in the potential to reduce the risk of developing dementia, and that public messaging will be strengthened by a robust supporting evidence base.

### A limited understanding of dementia hinders risk reduction research

There are fundamental gaps in the understanding of dementia that undermine the progress of risk reduction research. This is manifested in several ways. Firstly, there is a lack of consistency on the definition of the different dementias—whether to use neuropathological or clinical indicators—which in turn has significant impact upon a consensus of what constitutes primary prevention for dementia. Secondly, the current lack of validated biomarkers that may presage dementia represents a barrier, particularly when it may take decades from the onset of brain pathology for the clinical indicators of dementia to manifest.^12^ Thus, this means that studies that seek to measure the impact of behavioural or drug therapeutic interventions require forbiddingly long studies. Thirdly, there is a lack of understanding of how risk factors affect the pathophysiology of the disease and how they may be influencing the health of the brain and its ability to withstand the effects of dementia pathology.

#### Actions required

Strategies to prevent dementia should be a major theme in the research landscape. A change in thinking is required whereupon research into the causes, measurement and description of the pathophysiology of dementing diseases, including the study of neuropathological and clinical indicators and the continuum of change, is part of a spectrum that includes research into prevention of disease. This removes the somewhat arbitrary distinction between these fields and could lead to a more balanced portfolio of funded projects.

### Understanding the complex, life-course interplay of the risk factors

It is well recognized that dementia is a complex, multifactorial condition, with risk factors asserting varying influences throughout the life course. It has been argued that many of the published trials in dementia have considered the impact of a single intervention on a single risk factor, which is overly simplistic.^13^ The recent interim results from the FINGER study demonstrate that it is possible to have modest impact on cognitive decline through reductions in multiple risk factors.^14^ Given that some of the observed associations between risk factors and development of dementia may reflect early features of dementia, there is a real challenge to control for reverse causation. Without controlled, interventional studies, it is not possible to infer causality from risk factor associations and to know therefore whether behavioural modification will reduce the incidence, or delay the onset, of dementia.

#### Actions required

Funders should acknowledge the relative infancy of research into dementia risk reduction. In order to build the research base, there should be explicit acknowledgement of the length of time required to generate useful findings, which stretches beyond normal timeframes for research funding. There may also be scope to utilize life-course epidemiology methodology to reduce timescales and costs of research. It is also important to acknowledge the difficulty in unpicking the complexity and interplay between risk factors. Overcoming these factors requires more expertise, time and money. Given the modest research base, experience in the field is restricted to a relatively small group of people and encouraging or facilitating access to writing groups or expertise in study design or statistics, for example, may increase the prospects of success for grant applications in this area. Ensuring that peer reviewers have the insight to recognize the inherent challenges of the field will help to enable robust applications to progress.

### Variation in study design

A major barrier to progress of the risk reduction evidence base is the inconsistency of methodology, a lack of standardization of definitions, terminology and outcomes.^15^ This can hamper the pooling or comparison of data from different sources for additional analyses.^16^ Given the costs and long timescales associated with risk reduction studies, particularly randomized control trials (RCTs), it is frustratingly difficult to compare long-term studies due to the wide variation in inclusion and exclusion criteria, methodology, clinical instruments used, intervention and duration of interventions, observations and outcomes. These are lost opportunities. Progress has been made in this area within cardiovascular prevention research, for example, using large cohort studies to examine determinants and outcomes of cardiovascular risk factors in childhood.^17^

The inconsistency in the field reflects the broader issue of poor strategic overview of the research landscape. This has resulted in a lack of surveillance to capture the current prevalence of dementia and there are ongoing proprietary and technical issues around data sharing within the National Health Service and the research communities in the UK. At the World Health Organisation (WHO) Global Action Against Dementia meeting in March 2015 the WHO committed to setting up a ‘Dementia Observatory’ that may address this issue in due course and encourage greater consistency in definitions and registration.^18^

#### Actions required

National and international leadership and strategy is needed to provide coordination and a degree of standardization of research approaches. At a time of burgeoning interest in dementia risk reduction, the opportunities and resources available need to be used efficiently and effectively. The Joint Programme on Neurodegeneration (JPND) has convened a series of working groups on cohort studies to improve consistency in research design.^19^ There are also initiatives such as the NIH Toolbox^20^ in the USA, which is developing a set of consistent set of measures that can be used as a common currency across a range of research studies.

Leadership is required to develop prospective and retrospective standardization of data, to ensure consistency with existing studies both in the UK and internationally. Strategic oversight at international and national levels (e.g. JPND at a European level, the Medical Research Council (MRC) and the National Institute for Health Research (NIHR) in the UK) is crucial to ensure that data sharing for academic purposes is considered and supported appropriately by policy-makers. Current public mistrust of data sharing must be addressed to ensure that the unique data available through the National Health Service can be used as a powerful research tool.

There also needs to be support to extend or add-on to other existing risk reduction studies, in fields such as cardiovascular disease, to incorporate cognitive measures. Resources are available, such as the Dementias UK Platform, which offer opportunities for further development of study design and methodology.

### The challenge of applying RCTs to risk reduction research

The role of RCTs within risk reduction research elicits mixed views. For some, there is insufficient underpinning basic science to warrant undertaking expensive and time-intensive RCTs, particularly within the context of the multifactorial, life-course nature of dementia risk reduction. There is also a challenge of extrapolating from the controlled environment of an RCT to the real world to ensure the intervention is actually practical.^21^ For others, there is recognition that RCTs are the best way to determine causality and should be a priority for research investment in this field.

#### Actions required

For RCTs to generate meaningful outputs, tools to improve the scale, scope and linkage of data are needed: access to medical records and agreement on which measurable endpoints to use were identified as priorities. There is also a need to better utilize statistical modelling. One approach to consider for future risk reduction trials is to take a multi-modal, multi-factor, multi-level and individually tailored approach.^22^ A recent evidence review for dementia risk reduction^3^ outlined several areas where RCTs would be particularly informative, including late-life physical and cognitive activity and the impact of the management of diabetes on the incidence of dementia.

### Cultural barriers to collaboration and interdisciplinarity

A key component to meaningful progress is the fostering of collaboration between research groups both within the UK and internationally. There is a current perception that there is considerable focus on achieving outputs to support individual or institutional academic publications rather than long-term observational studies that need collaboration between multiple organizations. There are good examples of risk reduction research collaborations in mainland Europe such as HATICE,^23^ pre DIVA,^24^ FINGER^14^ and MAPT.^25^ These studies have recognized the importance of a solid basis of sharing experience and data, which from their perspective helped to facilitate funding.

#### Actions required

We look to all funding organizations to consider longer term models of funding that will support the particular needs of dementia risk reduction research. This could include increasing the number of funding schemes that explicitly provide the option of long-term funding in tranches, with clear milestones. It may also include working collaboratively with other funders to create specific, tailored funding streams. There are broader structural and cultural changes that would support the field to challenge the current disincentives such as support for grant writing.

The dementia research community can also learn from the experience of other fields, such as cardiovascular disease or cancer, where risk reduction research is more developed and research models could be translated and applied to the dementia field. This could include using developments in methodology, modelling, data analysis and broader lessons learnt.

### Lack of research capacity and leadership

There is agreement that the risk reduction research community is currently under-resourced relative to the potential impact and advances that could be made in the field. There are issues of collaboration between disciplines, and particular capacity problems within public health and clinical care to participate in risk reduction research.

#### Actions required

Given the complex interdisciplinarity required to develop the field, it is crucial that research capacity within a range of fields including public health, epidemiology and clinical dementia are supported and nurtured. There is a need to support junior investigators, as future research leaders, to develop the multidisciplinary methodologies and approaches required to overcome the identified barriers. This returns to the need for strategic national and international leadership to define both the research priorities and ensure that the researchers are available to deliver these priorities. Importantly, funding mechanisms should exist that overtly encourage cross-discipline collaboration.

### Stigma as a barrier to research

Public understanding of dementia risk reduction is currently very low. There are several reasons for this: firstly, the stigma of dementia and mental illness generally does not foster engagement;^26^ secondly, the evidence supporting the case that behavioural or environmental modification will reduce dementia risk on an individual basis has been perceived as too weak to support widespread public information dissemination or other behaviour change mechanisms; thirdly, population-level evidence has by definition largely been derived from commonest causes of dementia, namely sporadic AD and vascular dementia.^27^ However, the prevalence of mixed dementia means that there is often vascular and or AD pathology within many dementia patients whose primary diagnosis is neither AD nor vascular dementia.^28^ Consequently, dementia risk reduction has not been incorporated within non-communicable disease prevention strategies until the recent NICE public health guideline on midlife approaches to reduce dementia.^29^

#### Actions required

Engagement with policy-makers is needed to highlight the potential of dementia risk reduction and to support tailored approaches to public messaging. This in turn will help to make the case for greater research investment to enrich the evidence base. The research and wider dementia community needs to highlight the importance of participating in research and raise awareness of initiatives, like Join Dementia Research that encourage public participation in research.^30^

## Discussion

### Main finding of this study

The evidence base for dementia risk reduction is growing, albeit too slowly, and there is increasing political support that coincides with a greater focus upon the prodromal disease. However, if we are to make the shift from a small research field with a lack of a multifactorial causal analysis, we need to change the emphasis and scale of our approach to risk reduction research. The key objective for the research community is to provide clarity on the key risk factors and an understanding of effective interventions, such that this can be translated into effective public and professional messaging.

Researchers and funders working within the wider preventative health disciplines, such as obesity, diabetes management or physical activity, need to recognize the potential of their work to support dementia risk reduction. Such support could include funding calls specifying a requirement for longer term health outcomes, such as cognitive function, to be measured as part of any study.

The next steps required:

- National and international leadership around dementia risk reduction, to promote its particular needs and create integration with other fields.
- Funders to highlight opportunities and support for risk reduction research.
- Activities to promote collaboration and interdisciplinary working.
- Support for RCTs into risk reduction.
- A commensurate response from the research community with high quality applications in dementia risk reduction research.

### What is already known on this topic

The evidence base for dementia risk reduction has been growing in recent years,^3^ and the political acknowledgement of its potential to reduce incidence of dementia has occurred in the past 2 years.^8,31^ There is, however, recognition that there are gaps in the evidence base.^3,32^

### What this study adds

This work has enabled researchers and funders across the dementia risk reduction field to meet, discuss and prioritize the barriers and potential solutions to the issues associated with progressing research in this discipline. It has offered an opportunity to strategically assess how the field needs to develop in the future to address the recognized evidence gaps as quickly as possible.

### Limitations of this study

The findings of this paper are based on discussions of a 1-day workshop with 58 invited attendees. Inevitably time constraints limit the potential to consider all the issues and the topics of discussion depend on the particular views expressed. However, the invited attendees represented all the key disciplines in the field and there was broad consensus around the identified priority findings, which are presented in this paper.
